# From Beethoven to Beyoncé: Do Changing Aesthetic Cultures Amount to “Cumulative Cultural Evolution?”

**DOI:** 10.3389/fpsyg.2021.663397

**Published:** 2022-02-09

**Authors:** Natalie C. Sinclair, James Ursell, Alex South, Luke Rendell

**Affiliations:** ^1^Centre for Biological Diversity, School of Biology, University of St Andrews, St Andrews, United Kingdom; ^2^Department of Philosophy, University of St Andrews, St Andrews, United Kingdom; ^3^Royal Conservatoire of Scotland, Glasgow, United Kingdom

**Keywords:** cumulative culture, cultural evolution, aesthetic value, music evolution, animal culture

## Abstract

Culture can be defined as “group typical behaviour patterns shared by members of a community that rely on socially learned and transmitted information” (Laland and Hoppitt, 2003, p. 151). Once thought to be a distinguishing characteristic of humans relative to other animals (Dean et al., 2014) it is now generally accepted to exist more widely, with especially abundant evidence in non-human primates, cetaceans, and birds (Rendell and Whitehead, 2001; Aplin, 2019; Whiten, 2021). More recently, cumulative cultural evolution (CCE) has taken on this distinguishing role (Henrich, 2015; Laland, 2018). CCE, it is argued, allows humans, uniquely, to ratchet up the complexity or efficiency of cultural traits over time. This “ratchet effect” (Tomasello, 1994) gives the capacity to accumulate beneficial modifications over time beyond the capacities of a single individual (Sasaki and Biro, 2017). Mesoudi and Thornton (2018) define a core set of criteria for identifying CCE in humans and non-human animals that places emphasis on some performance measure of traits increasing over time. They suggest this emphasis is also pertinent to cultural products in the aesthetic domain, but is this the case? Music, art and dance evolve over time (Savage, 2019), but can we say they gain beneficial modifications that increase their aesthetic value? Here we bring together perspectives from philosophy, musicology and biology to build a conceptual analysis of this question. We summarise current thinking on cumulative culture and aesthetics across fields to determine how aesthetic culture fits into the concept of CCE. We argue that this concept is problematic to reconcile with dominant views of aesthetics in philosophical analysis and struggles to characterise aesthetic cultures that evolve over time. We suggest that a tension arises from fundamental differences between cultural evolution in aesthetic and technological domains. Furthermore, this tension contributes to current debates between reconstructive and preservative theories of cultural evolution.

## Introduction

Culture can be broadly defined as “group typical behaviour patterns shared by members of a community that rely on socially learned and transmitted information” (Laland and Hoppitt, 2003, p. 151). Cultures evolve, in the sense that they change over time, and there is vigorous and ongoing debate over the extent to which this cultural evolution can be understood in the same or similar Darwinian framework that underpins our understanding of genetic evolution (Mesoudi, 2011; Claidière et al., 2014; Nettle, 2020; Rosenberg, 2021). Cultural transmission occurs through different social learning pathways: vertically (parent to offspring), horizontally (between individuals of the same generation), or obliquely (between unrelated individuals of different generations) (Cavalli-Sforza and Feldman, 1981). There is, however, disagreement as to whether this transmission is dominantly preservative or transformative. Of course, preservation and transformation must both be present if culture is to evolve at all (see e.g., Gabora and Tseng, 2017), but debate about relative importance persists. Cultural evolutionary theorists view cultural transmission as preservative, in which variants are faithfully transmitted between individuals (with some degree of error). On the other hand, cultural attraction theorists argue that cultural transmission is reconstructive, wherein cultural variants are potentially transformed in the context of being reconstructed by the receiver (Mesoudi, 2011; Acerbi and Mesoudi, 2015; Scott-Phillips et al., 2018).

Human life is rich with culture pervading science, technology, customs, beliefs, art, literature, and music. Culture was once thought to be a distinguishing characteristic between humans and other animals (Dean et al., 2014) but is now generally accepted to exist outside humans, with evidence in non-human primates, cetaceans, and birds (Rendell and Whitehead, 2001; Whiten, 2011; Aplin, 2019). Although semantic disagreements persist (Heyes, 2020), there is ample evidence that the content of non-human culture evolves in the sense of changing over time (e.g., Garland et al., 2011). Despite this evidence from across the animal kingdom, there still appears to be something distinctive about the way human culture builds upon itself over time to increase the performance of our cultural products. This process, referred to as cumulative cultural evolution, or CCE henceforth (Boyd and Richerson, 1996; Tomasello, 1999), has become a primary focus for those trying to understand the differences between human and non-human culture, and how human populations collectively improve their cultural toolkits. Humans are able to “ratchet up the complexity or efficiency of cultural traits over time” through this process (Tomasello, 1994, p. 312; Tomasello, 1999).

If CCE is to be a feature of human uniqueness, then we need very clear ideas of what it is, and what it is not (Vaesen and Houkes, 2021). Our purpose here is to highlight what we see as an ambiguity in current thinking on the key features of CCE when it comes to cultural traits that are valued primarily or exclusively for their aesthetic properties – what we will term as “aesthetic cultural traits” or “aesthetic products”.

Using interdisciplinary perspectives on the philosophy of aesthetics, musicology, cultural evolution, and biology, we show here how the question in our title is not trivial, and that its answer will have important implications for how we think of CCE in humans and non-humans alike, using musical performance and non-human animal song as our principal motivating examples. One of our primary goals is to build bridges between a number of disciplines whose interests we see as overlapping on this question. Because of this, some material may be familiar to some readers but new for others, and while we do not pretend to provide comprehensive reviews of each area, we hope most readers interested in this general topic will find something informative from a discipline different to their own background.

Here we begin with an introduction to cultural evolutionary theory and ask how aesthetic cultures may fit into the current framework of CCE. We then examine whether aesthetic attractiveness (in terms of aesthetic value) can be measured sufficiently to enable its incorporation into this framework. We then take an example of an aesthetic culture – music – and explore whether this can improve over time. Finally, we discuss a case study of potential CCE in non-human animals – humpback whale song – through the lenses of these arguments. Our discussion is born of a realisation that we cannot evaluate whether humpback whale song is CCE without first determining how human aesthetic cultures fit into the CCE framework.

## Cumulative Cultural Evolution

Mesoudi and Thornton (2018) sought to define a set of core criteria for CCE in human and non-human animals. The core criteria comprise four steps or qualities:

i That behavioural variation exists.
ii A behavioural variant is passed onto others by social learning.
iii That the learned behavioural variant must *enhance some measure of performance* (our emphasis), and lastly.
iv That steps i, ii, and iii are repeated to create sequential improvement over time.

Recent literature is ambiguous regarding whether Cumulative Technological Culture (CTC) is merely one form of, or is synonymous with, CCE (Miton and Charbonneau, 2018; Osiurak and Reynaud, 2019; Osiurak et al., 2020). Mesoudi and Thornton (2018) sought to clarify the concept of CCE in part due to the diversity of definitions of CCE in the literature. They contemplated 35 definitions, of which eight specified technology in CCE. Mesoudi and Thornton’s conception of CCE is not however restricted by definition to the technological domain and could, theoretically, include any cultural trait which meets their core requirements. It is their requirement for improved performance that we focus on here. Examples of a performance measure may be “the efficiency of migratory routes or extractive foraging, the durability and sharpness of cutting tools, or *the aesthetic attractiveness of art or dress styles* (our emphasis).” (Mesoudi and Thornton, 2018, p. 2; note that Supplementary Tables 1A,B give further examples of performance measures used in the literature). However, while “aesthetic attractiveness” is mentioned as a performance measure early in their manuscript, it is not explored further. Our intention here is to pull at this thread, because the interdisciplinary discussions that led to the present analysis suggest it is not straightforward to say that aesthetic “attractiveness” can increase in a measurable way. In our discussion of this problem, we interpret aesthetic attractiveness to mean “aesthetic value” as used in literature in the philosophy of aesthetics and focus on that value as the experience of an aesthetic product, in context, by individuals.

We define aesthetic cultural traits as those which are created, transmitted, and consumed because of the intrinsically valuable experiences which sustained appreciation of them affords. Examples of these aesthetic cultural traits are primarily found in the arts, where traditional categories include (but are not confined to) those cultural products (or artworks) found within visual art, sculpture, literature, poetry, music, performance art, theatre, film, dance, and architecture (what we refer to as “aesthetic products”). We use the term “aesthetic culture” to refer to cultural activities and products (including events such as musical performances or live theatre) that have been designed to afford aesthetic experience or be objects of aesthetic appreciation. The concept of an “aesthetic domain” may seem nebulous, as almost any object, activity or process could be experienced aesthetically (as emphasised in the burgeoning “everyday aesthetics” literature – see Leddy, 2012; Melchionne, 2013; Davies, 2015; Saito, 2017), but we use the expression to refer primarily to the examples above, whilst accepting that the concept has a fuzzy boundary and can be applied to non-standard cases. We also note that although there is some philosophical scepticism regarding whether the different arts share properties which would allow them to be united into a single group (Kivy, 1967), we focus on examining aesthetic products as a whole, in the sense defined above.

Some cultural evolutionists maintain that an additional essential criterion of recognising cumulative culture is that no one individual would be able to create the behaviour, skill or knowledge in question on their own, such that the cultural product “is beyond the capacities of a single individual” (Sasaki and Biro, 2017)^1^. This is a point of contention within the literature between the “process” vs. “product” oriented views of cumulative cultural evolution (Reindl et al., 2020). Product oriented views assert *as a diagnostic criterion of CCE* that cultural products must be beyond the capacity of a single individual to create *de novo*. On the other hand, process focussed views emphasise the processes of iterated innovation and transmission that resulted in a given cultural product. If, for example, a group produces stone tools following a history of repeated learning cycles, as in Mesoudi and Thornton’s (2018) core criteria, it is an example of CCE irrespective of whether another individual in a different group at some point develops an identical stone tool *de novo*. A product-oriented definition presents some issues in the aesthetic domain however – anyone can invent a new tune, but would we consider the same sequence of notes differently if it had been produced by a babbling toddler rather than an advanced music student who had been trained in composition and its history? We need not be derailed by this debate here, since both views are reliant on the concept of an “improvement of performance” (or “ratcheting”), which forms the central concern of this article.

We think it is imperative for cultural evolution researchers to interact with disciplines that have existing traditions of thought and study related to the phenomena they are bringing under the cultural evolution lens, so our motivation here was partly to explore via interdisciplinary dialogue what it might mean to talk of cumulative cultural evolution in the aesthetic domain. Has art improved in the way that our capacity to reach celestial bodies has? While surely few would doubt the excellence of both in their respective contexts, is the music of Beyoncé really the product of countless iterations of *performance improving* innovation since the time of Beethoven? A principal reason to undertake this enquiry is that the resolution of this question has important implications for thinking about whether non-humans have elements of CCE, which we address through the example of humpback whale song. Mesoudi and Thornton (2018) are clear that they consider those behaviours transmitted by social learning that are fitness neutral as non-cumulative. They posit examples of first names in humans and changes in birdsong as showing neutral drift as opposed to cumulative evolution (Mesoudi and Thornton, 2018). But where is the line between “fitness neutral” and “aesthetic” to be drawn? If we are unable to determine how aesthetic cultures “improve” and are therefore cumulative, must we also consider large tracts of aesthetic human cultural products as the result of neutral drift as opposed to any kind of cumulative evolution? A secondary reason is that through our dialogues we have come to the view that current debates in cultural evolution between advocates of “traditional” approaches and more recent contributions from supporters of cultural attraction theory might be clarified by considering the way in which CCE might occur and/or differ in the aesthetic domain.

Borrowing terminology from Sterelny (2017) for efficiency, cultural evolutionary theorists of the “Californian” (“traditional”) (e.g., Boyd and Richerson, 1996; Acerbi and Mesoudi, 2015; Mesoudi and Thornton, 2018; Buskell, 2019) and “Parisian” perspectives (e.g., Claidière et al., 2014; Morin, 2016) agree that humans’ ability to live and thrive in a wide variety of ecological conditions is dependent on the accumulation of cultural learning over time, but they disagree about the relative importance of transmission versus construction in that process (Sterelny, 2017):

*The Californian perspective* (sometimes presented as the “traditional” view) frames cultural transmission as a preservative mechanism in which variants are chosen and faithfully transmitted between individuals (with some error) which creates overall stability in cultural traits across time (Acerbi and Mesoudi, 2015).
*The Parisian perspective*, specifically cultural attraction theory (CAT), emphasises transformative processes in which cultural variants are reconstructed by the receiving individual. CAT aims to explain cultural variation by way of cultural attractors. Cultural attraction theory includes the concept that some variants are statistically more likely to be reconstructed due to inherent biases within the individuals doing the reconstruction (Morin, 2016).

Proponents of the Californian perspective question the validity of CAT as a separate theory to explain culture (Buskell, 2017a,b, 2019), but Acerbi and Mesoudi (2015) assert that these two theories are not necessarily in contrast to each other, arguing a broad cultural attraction theory may encompass the same processes addressed by cultural evolutionary theory; in contrast CAT proponents defend the distinctness of their framework (Morin, 2016; Scott-Phillips et al., 2018)^2^.

Is this debate an unresolvable clash between two fundamentally different views of cultural evolution, or do the different perspectives arise because they are primarily focussed on fundamentally different forms of cultural evolution – consistent with Vaesen and Houkes (2021) we use the term “technological” cultural knowledge (e.g., how to build canoes) in the Californian case, as opposed to forms of culture that operate more exclusively in the aesthetic domain (e.g., heraldic symbols)? We will argue that appreciating the fundamental differences of what it means to talk about CCE in technological and aesthetic cultural contexts lends support to Acerbi and Mesoudi’s (2015) assertion that these schools can co-exist, since their ideas originate in fundamentally different types of cultural evolution. In the “technical” realm, it is unproblematic to think about ratchetting improvements, and to those improvements being transmitted, and tested against an external environment. In the aesthetic domain however, psychological processes like cultural attraction will increase in influence, as the form of the cultural products is not tested against an external environment, but more by the experience of viewing or listening to them, and the responses evoked therein. Here, the aesthetic process has much more in common with the transformative accounts of CAT, but as we shall see, it is more problematic to think about an aesthetic “ratchet.”

## Can Aesthetic Value Improve Cumulatively?

Mesoudi and Thornton (2018) propose “aesthetic attractiveness” as one measure of performance that could show cumulative improvement. Their prospect of measuring aesthetic attractiveness intersects with topics in philosophical aesthetics – specifically, the subjectivity of taste – which we discuss in this section. We interpret “aesthetic attractiveness” here as “aesthetic value,” since the latter has more currency in the aesthetics literature. However, the conclusion we motivate, that aesthetic value may lack the objectivity needed to be a good proxy for the improvement that is a core criterion of cumulative cultural evolution, is equally applicable to “aesthetic attractiveness.”

What, then, is “aesthetic value”? The most common view in the philosophical aesthetics literature states that an object has aesthetic value or disvalue by virtue of, and in proportion to, the quality of the aesthetic experience it can produce in spectators who meet standard viewing (or listening, tasting, smelling, and so forth) conditions (see for example: Munro, 1955; Beardsley, 1958, p. 333; Watkins and Shelley, 2012, p. 531; Stecker, 2006, p. 5). Standard viewing conditions specify minimum conditions which a percipient (a person who is able to perceive things) must meet for her aesthetic experience of an object to be representative of the calibre of aesthetic experiences which that object can produce. Examples of standard viewing conditions include having functioning sensory and cognitive capacities, having art historical or contextual knowledge, as appropriate, about the work or object (or performance) to which they are attending (such as the knowledge of a painting’s provenance and the ability to classify it in the correct genre), and having experience of suitable comparators^3^.

According to this view, hereafter “the standard model,” an artwork or aesthetic product which consistently produces enriching, satisfying or rewarding aesthetic experiences is aesthetically valuable for doing so. Conversely, a work which elicits dull, onerous or nauseating aesthetic experiences thereby has aesthetic disvalue. The standard model casts aesthetic value as a kind of instrumental, rather than final, value (where instrumental value is the value something has a means to an end, and final value is autotelic; the value something has as an end or “for its own sake”): aesthetically valuable objects are valuable because they are means to aesthetic experiences.

This raises the issue of what makes an experience “aesthetic.” Aesthetic experiences vary in their duration, intensity and character. Some are brief moments of fleeting pleasure in which we savour a sumptuous quality we chance upon in our surroundings: the fragrant scent of a plant, or the undulating peal of church bells. Other aesthetic experiences are not so pleasant: we may feel repulsed, oppressed, distressed, indignant, or frustrated. Think, for example, of a formulaic pop song played *ad nauseum*, or the decomposing carcass of a bird. Francis Bacon, the painter, alludes to aesthetic experience having a restorative effect in the following:

“If I go to the National Gallery and I look at one of the great paintings that excite me [.] the painting unlocks all kinds of valves of sensation within me which return me to life more violently” (Sylvester, 1987, p. 141).

The intensity of some aesthetic experiences can displace the sense we have of ourselves as experiencing subjects who are apart from the observed world. These experiences may acquire a quasi-spiritual or quasi-religious character. Ralph Waldo Emerson describes one such experience in the following:

“I see the spectacle of morning from the hilltop over against my house, from daybreak to sunrise, with emotion which an angel might share … the active enchantment reaches my dust, and I dilate and conspire with the morning wind” (Emerson, 2003, p. 43).

Attempts by philosophers efforts to explain what makes these experiences “aesthetic” can be situated in four camps: (i) the content-oriented approach which characterises aesthetic experiences in terms of the qualities at which they are directed (see Carroll, 2002, 2006, 2012, 2015); (ii) the axiological approach which treats aesthetic experiences as being of final, and not just instrumental, (dis)value (see Stecker, 2001, 2005; Iseminger, 2006); (iii) affect-oriented approaches, which characterise aesthetic experience in terms of a distinctive affective state, set of affective states or a type of pleasure (e.g., Bell, 1914/1987; Beardsley, 1969); and (iv) attitudinal accounts, which explain aesthetic experience by reference to an “aesthetic” attitude or a way of allocating attention (see Stolnitz, 1960; Bullough, 2008; Nanay, 2016, 2018). Hybrid positions which combine several of these approaches are also possible.

We now have a rough outline of what Watkins and Shelley (2012) describe as the ‘‘dominant’’ view of aesthetic value^4^. Thomas Munro expresses it in the following:

“Works of art as products – pictures, poems, and sonatas – can be good only instrumentally, as means to good experience in someone at some time […] No work of art or “objective” quality in art (such as unity or balance) can be good in itself […] It has aesthetic value as a means to good aesthetic experience” (Munro, 1955, p. 333).

Monroe Beardsley puts it in slightly different terms:

‘‘X has aesthetic value’’ means ‘‘X has the capacity to produce an aesthetic experience of a fairly great magnitude^5^ (such an experience having value)” (Beardsley, 1958, p. 531).

More recently, here is Robert Stecker:

‘‘Aesthetic value comes in two varieties. There is the intrinsic value of aesthetic experiences themselves by which I just mean that they are valuable in themselves. There is the instrumental value of objects capable of delivering aesthetic experience to those who understand them’’ (2006, p. 5)^6^.

**FIGURE 1:**
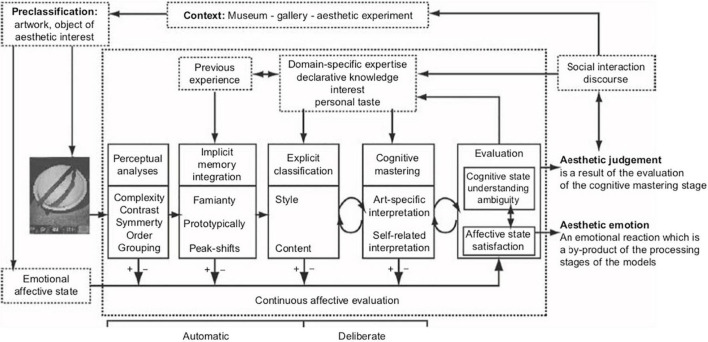
Taken from Leder et al. (2004): A model of aesthetic appreciation and aesthetic judgement.

If aesthetic value is to be a proxy for cumulative improvement, as Mesoudi and Thornton suggest, then we need to be able to measure it in some way. The standard model gives us a rough sense that this would involve examining the quality of aesthetic experiences an object affords a subject who meets standard viewing conditions. However, it is unclear how much consensus there could be about aesthetic value which is measured in this way. A given object may afford different aesthetic experiences for different people depending on their tastes and preferences (which have themselves many inputs including from the individual’s expertise, cultural background and the context in which an object is presented – see Figure 1). Consider Death Metal music. A piece of Death Metal may provide an intensely satisfying aesthetic experience for one person and a torturous and unpleasant aesthetic experience for another. The amount of aesthetic value or disvalue the piece has would therefore seem to depend on *whose* aesthetic experience we study.

If, as the standard model implies, the aesthetic value of objects derives from our aesthetic *experiences* of them, and those experiences vary, then we may have to index measurements of aesthetic value to individual percipients. Quite how much consensus or divergence there is amongst aesthetic experiences could be established through empirical research (by, for example, conducting surveys). It seems at least probable that we could identify trends that indicate which artworks provide high quality aesthetic experiences and which do not. What remains unclear is how much consensus would be needed in order for measurements of aesthetic value to be a good proxy for the improvement as a criterion for CCE.

Clearly, an argument for classifying a behaviour as CCE would seem, at least, less compelling if there is a lack of consensus about whether the products of that behaviour had improved by a measure of performance. To this concern, we can offer four responses that warrant further consideration:

1. Accept that measurements of aesthetic value vary relative to the tastes and preferences of individual percipients and accept that that putative cases of CCE which advert to aesthetic value are on shaky foundations; [indeed, some implicitly take this approach by focussing specifically on “cumulative technological culture” as the explanandum (Osiurak and Reynaud, 2019)].
2. Argue against relativism, which David Hume famously does^7^ (see Hume, 1757/1995), though not without facing considerable challenges [see Kivy, 1967; Shusterman, 1999; Levinson, 2002, p. 229].
3. Argue that there is too little consensus about aesthetic value to justify using measurements of it as a proxy for cultural fitness.
4. Argue against the standard model of aesthetic value and in favour of an alternative which is less vulnerable to objections from relativism (though it is unclear what such an alternative would look like).

In summary, the standard model provides a framework within which aesthetic value can be empirically investigated; we can measure the aesthetic experiences of people who satisfy standard viewing conditions and, in doing so, learn how aesthetically valuable the objects of their experiences are. However, it remains to be seen how much intersubjective validity measurements of aesthetic value gathered in this way could have. CCE requires a “ratcheting” of improvements in some measure of performance over time. But how much intersubjective validity do these measures of performance need? Further research could establish just how objective a measure of performance needs to be for CCE and just how much or little consensus there is among our aesthetic experiences.

## Does Music Improve Over Time?

Moving from general considerations of improvement in aesthetic value, we now consider how concepts of improvement and progress have been deployed in a highly significant area of human aesthetic culture – music. Mesoudi and Thornton (2018) do not expand upon their identification of “aesthetic attractiveness of art” as a possible measure of performance. Contrastingly, Mesoudi (2011) posits that the sort of change seen in the aesthetic aspects of music is not a matter of improvement but is better captured by the notion of cultural drift which is the result of the random copying of cultural variants. We investigate this tension now, firstly looking at how “progress” (including the sense of increasing aesthetic value) has been theorised in historical musicology and ethnomusicology. We consider technical advancements and other developments which some authors argue lead to “local” improvements in aesthetic value. Lastly, we review empirical studies utilising large digital datasets.

In 1788 Johann Nikolaus Forkel, a founding figure in modern historical musicology, invoked a striking image of an octopus (Forkel, 1788, translation in Dahlhaus, 1987). He used this image to capture his vision of the teleological development of ‘‘the arts and sciences [which] only grow to *perfection* gradually,’’ in ‘‘stepwise *progression* from the simple to the complex’’ (our emphasis)^8^.

Nearly two hundred years later in an essay on “Progress and the avant garde,” musicologist Carl Dahlhaus (1987) referred back to Forkel’s octopus to illustrate “the paradox of the idea of progress.” This paradox arises when we take a particular view on musical change: that music is inexorably developing through a series of “stages,” becoming more complex and, in some sense, improving. Although this idea has not survived detailed examination of the world’s musical cultures (Nettl, 2006), it seemed like a certainty to Forkel, no doubt nurtured by living in the optimistic Enlightenment, and continued to be a common implicit assumption and explicit declaration well into the twentieth century, by which time it was being illegitimately backed up by misapplied arguments taken uncritically from Darwinian evolutionary theory (Mundy, 2006, 2014).

This assumption of progress – or growth – toward perfection runs counter to a second commonly held intuition: that the acknowledged masterpieces of a particular style or period are not superseded by subsequent works. Stravinsky’s “Rite of Spring” is no higher in aesthetic value than Beethoven’s “Eroica,” Radiohead’s “Kid A” no improvement on The Beatles’ “Abbey Road.” As Dahlhaus puts it, it would “be blindly presumptuous to ascribe a higher rank to the musical present than to the past.” In this context he cites music historian François-Joseph Fétis, who appealed to the view that the goal of music is emotion, and wrote against the prevailing mood of his day that “in general what we call *progress* is only *transformation*…’’ (1835, our translation)^9^. “Change rather than progress” with respect to aesthetic value is currently the conventional view in musicology.

However, there may be progressive development in various aspects of musical *means* and “language.” Such improvements in the technological means (or means of production) through which musical works are produced and performed have occurred and are generally gradual (e.g., the nineteenth century expansion of the Western orchestra both in size and variety of instruments) but some are customarily viewed as revolutionary (e.g., the rapid uptake of staff notation in Gregorian Chant, or the introduction of digital production to popular music).

Regarding musical language, the discovery of harmony has often regarded as a key stage in the development of music (e.g., Spencer, 1890; Gilbert, 1920; Benzon, 1993), enabling wholly new domains of aesthetic experience. We might also listen to those composers and musicologists who have seen progress in terms of the development of a musical language, or in the way of *thinking through music*, as the addressing of certain “technical puzzles” (Adorno, 2020). Dahlhaus describes this process with respect to the music of Stockhausen:

“Difficulties which at first seemed insoluble provided the stimulus for works at a second level on which earlier problems were solved. Admittedly, others arose in their stead, but these in turn urged musical thinking onward. This seems to suggest that musical development in a restricted sphere, that of compositional technique, shares certain traits with the progress of a scholarly discipline” (1987, p. 20).

However, importantly, we should strongly resist the idea that there is any single or privileged musical language. Even within Western Art Music (WAM), the past century has arguably seen the end of the so-called “common practice period,” a strong and largely successful challenge to the hegemony of musical modernism, and the emergence and spread of multiple and very disparate styles (new complexity, minimalism, neoclassicism, neoromanticism, and spectral, etc.). If there is “progress” within a language it is severely local (e.g., we might think of how Schoenberg’s serialism with respect to pitch was broadened into “total serialism” in the works of Boulez and Stockhausen), and certainly cannot be measured in any absolute terms. Returning to our question: “Does music improve over time?” it is surely undeniable that gradual and sometimes ratchetting, in the sense of being very unlikely to be reversed, development of this kind result in changes in aesthetic experience and hence aesthetic value. Nonetheless, the history of the rise and fall of classical and popular musical styles, and the changes in popularity of individual artists, demonstrates the wide range of the evaluation of such changes and offers no support for global or unilineal increase in aesthetic value.

In comparative musicology and ethnomusicology, progress was problematised from the mid-1920s onward, particularly after World War II (Mundy, 2006, 2014). An example of this rejection is found in Curt Sachs’s (1961) posthumously published “The Wellsprings of Music” in which he describes progress as a “dangerous slogan,” and writes that “[w]e no longer believe in a neat evolution from low to high, a constant development from unassuming simplicity toward an ever growing complication.” He criticises the internal contradictions of teleological views of musical history with the telling point that their adherents frequently held up an earlier period as offering the most perfect music. Leonard B. Meyer echoes Sachs in his “Music, The Arts, And Ideas” (1967), in which “the demise of the idea of progress” in music is seen as part of a wider social and historical movement. Meyer argues that “[w]ith the development of historical musicology and ethnomusicology, the notion of stylistic progress has to all intents been given up.” Nonetheless Sachs still recognises a form of limited progress, in which each period sets “for art a temporary goal of its own,” a goal which may require the acquisition of new techniques and new means of expression. Sachs finds musical progress in the early development of opera, and the changing treatment of recitatives from Peri to Monteverdi. “Progress exists at best within a limited span; as to the total of art, there is no progress, no regress, but simply otherness.”

Meyer (1967) offers perhaps the most systematic and comprehensive account of stylistic change in general, which although focussed on the history of WAM is broad enough to include other musics. Alongside the “apparently random” changes that have been the focus of some contemporary modelling studies (e.g., Bentley et al., 2007) and which we discuss below, Meyer also discusses “mutational change.” This is particularly relevant to us because such revolutionary changes (such as the discovery of linear perspective in the visual arts, serialism in music, or the invention of new aesthetic goals) are said to give rise to “permanent and fundamental alteration” in the “fundamental presuppositions” or “premises” of a style. In their irreversible effects such paradigm shifts resemble the operation of Tomasello’s ratchet and could be linked to Sachs’s views on limited aesthetic progress. Once new premises have been established, artists work to explore the new realm of aesthetic possibilities offered by the new technological means, musical forms, or aesthetic goals. Meyer argues that the resulting period of intra-stylistic change is best captured by a model where change is predominantly driven internally rather than externally and is typically (though not inevitably) associated with a growth in complexity and reduction in informational redundancy.

Turning from historical musicology to the empirical sciences, the development of computational techniques in the field of Music Information Retrieval (Lartillot et al., 2008; Schedl et al., 2014), coupled with the assembling of large digital archives of recorded music and databases such as the Million Song Dataset (Bertin-Mahieux et al., 2011), has made it more straightforward to pose testable scientific hypotheses on various aspects of the cultural evolution of musical styles (Brand et al., 2019). Although it is true that some published studies using “evolution” in their title either do not use concepts or tools informed by evolutionary biology, instead they indicate a quantitative analysis of temporal trends and patterns (e.g., Serrà et al., 2012); or use biological measures of population change (such as diversity and disparity) without attempting to account for their causes (Mauch et al., 2015), there is also a significant body of research addressing whether such changes can be better explained through cultural drift alone (resulting from random copying) or when coupled with transmission or psychological bias (the term used to capture the effects of listener preferences, whether determined by musical features, desire for novelty, or social pressure to conform) (Acerbi and Bentley, 2014).

To raise the possibility that trends in musical cultures may be explained without reference to listener preferences is in some ways to question the very possibility of aesthetic progress and seems to strike at the notion of meaningful agency on the part of both music creators and audiences. Yet support for this possibility has arisen from corpus studies into the ability of a random copying model versus models incorporating transmission biases to predict observed turnover rates of songs in album and internet charts (Bentley et al., 2007; Acerbi and Bentley, 2014), turnover rates in the frequency of use of drum samples (Youngblood, 2019), and the dependence of the changing emotional content of lyrics on content and model biases (Brand et al., 2019). This research suggests that chart trends can predominantly be explained through cultural drift, with some evidence for conformity bias for specialist genres (Acerbi and Bentley, 2014; Youngblood, 2019), and a content bias for negative lyrics (Brand et al., 2019).

On the other hand, the rather unintuitive conclusion that chart success is mainly the upshot of random copying and has little to do either with its aesthetic value or with the content bias of the listener (i.e., a preference based on aesthetic experience), is challenged by other work which shows that success can be well-predicted through acoustic properties (Interiano et al., 2018), and is influenced by various measures of musical complexity (Percino et al., 2014; Parmer and Ahn, 2019). Moreover, moving outside the realm of Western pop music, Nakamura and Kaneko (2019) have demonstrated that trends in dissonance across four centuries in Western classical music can be reproduced in a simple evolutionary model excluding random copying, in which creators learn from the past and evaluators make selections based on novelty and style conformity; and further, that this simple model successfully predicted changes in an unrelated genre.

Finally, another perspective on this debate is provided by the results of the “DarwinTunes” experiment reported by MacCallum et al. (2012). Here, a “population” of short melodic loops, with successive generations being generated through modelled random mutation and reproduction, was allowed to evolve under the pressure of selection governed by listener preference. Once again, a balance is struck between cultural drift and psychological bias. It is striking that harmonic and rhythmic properties of the loops approached those commonly considered aesthetically pleasing in Western pop music: i.e., it appears that listeners chose tunes based on aesthetic grounds rather than at random. It is difficult to compare the different contexts offered by this experiment and the corpus studies into real-world music-buying habits described above, but the results of MacCallum et al. (2012) are consistent with iterated learning experiments showing that learning biases in the copying of drum patterns leads quickly to “rhythmic universals” (Ravignani et al., 2016). Together these studies may offer comfort for those seeking to hold on to a notion of agency. In a comment on the DarwinTunes experiment from the perspective of the “Parisian” perspective of cultural evolution, Claidière et al. (2012) emphasised the importance of guided transformative processes rather than random mutation in the evolution of “real music.” To us, this points to the need to take into account the makers of music as well as its audience, and the combined message is that creation and choice may after all be a driving force in cultural evolution. We note that in the artificial context of the “DarwinTunes” experiment the proxy of mean listener preference is used, and as we have unpacked in our philosophical analysis of aesthetic value above it is unclear whether preference can equate to a measurement of aesthetic value. It is also difficult to extrapolate from the results of this experiment context to the way in which the world’s diverse musics have altered over time.

Over the course of the last hundred years, historical musicology and ethnomusicology have come to the conclusion that any notion of global aesthetic progress is dead in the water, inescapably bound up with discredited social Darwinist notions of cultures evolving toward some idealised Western pinnacle. Nonetheless, in addition to undeniable technical and technological advancements, there are some strictly limited and local cases in which we might speak of improvement: Dahlhaus’s advances in “musical thinking” within a specific musical language, Sachs’s temporary progress toward particular aesthetic goals requiring the development of new techniques, and Meyer’s exploration of the possibility space of a new style. Empirical support for these limited cases may come from the corpus studies discussed above, which have demonstrated an increase in instrumental complexity associated with the growth of new popular music styles (Percino et al., 2014), and have confirmed the increased use of dissonant harmony in the history of WAM (Nakamura and Kaneko, 2019). However, the quest for, let alone the identification of, a culture-independent measure of global “aesthetic value” has long been abandoned in musicology, and the empirical studies cited have instead used proxies of chart success or listener preferences. Aesthetic goals, when considered at all, are seen to be learned, set and evaluated from within particular musical cultures. Each musical culture can and perhaps should be thought of as a distinct stem of a constantly diversifying evolutionary bush rather than steps on a ladder. Adopting this perspective, who is to judge the relative merits of the musical productions of a Beethoven and a Beyoncé?

## Is Cumulative Cultural Evolution Unique to Humans?

While students of philosophy and musicology may be familiar with the preceding content, it may not be so obvious why it could, as we argue now, be relevant to debate at the interface of human and non-human animal cultural evolution. If CCE is to be somehow diagnostic of human uniqueness, then there will inevitably be great interest in understanding whether anything like it occurs in non-humans. Some have challenged the claim that CCE is unique to humans. For example, Hunt and Gray (2003) posited tool manufacture in New Caledonian crows (*Corvus moneduloides*) as CCE. A variety of cultural behaviours in primates have also been postulated as cumulative in character from nutcracking behaviour in chimpanzees to eye-poking in capuchin monkeys (Cebinae) (Perry, 2011), More recently, Jesmer et al. (2018) have shown evidence for the CCE of migration routes in relocated populations of both bighorn sheep (*Ovis canadensis*) and moose (*Alces alces*). Individuals from a population of bighorn sheep that had been established in the environment for over 200 hundred years were found to have double the efficiency in their migration route compared to individuals of a population that had only been established for up to 35 years. This was due to a longer history of repeated cycles of innovation (in movement decisions) and learning, very similar to Mesoudi and Thornton’s (2018) core criteria, in the longer established populations.

**FIGURE 2:**
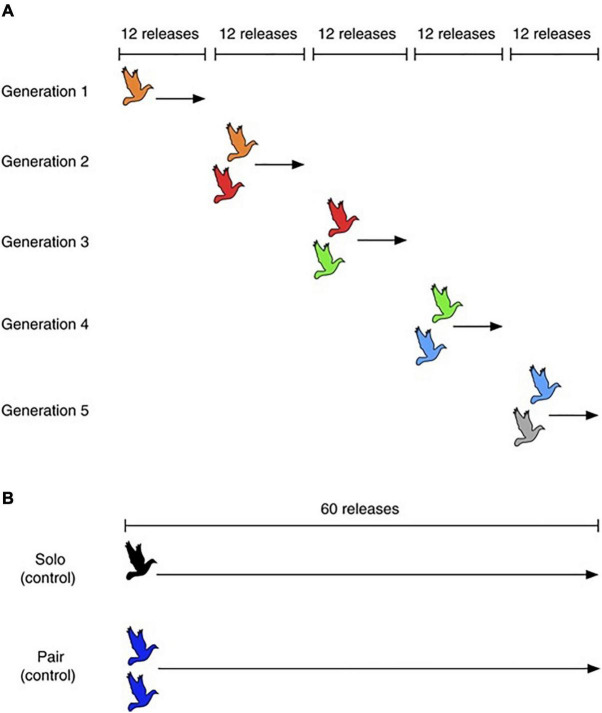
From Sasaki and Biro (2017): “Homing flight release protocols. **(A)** Experimental group and **(B)** control groups. In each chain of the experimental group, a single pigeon (orange) was first released from the same site repeatedly 12 times, then partnered with a naive pigeon (red) and flown as a pair a further 12 times. The first bird was then replaced by a third bird (green) and this new pair (red þ green) was also released 12 times. This procedure continued until the fifth-generation bird (grey) was added and flown a final 12 times. In the control groups **(B)**, single pigeons and fixed pairs were released the same number of times as the total flown by the experimental group (60 flights). All three treatment groups contained 10 independent replicates (chains, solo birds, or pairs)”.

Further provocative evidence for CCE in non-human animals comes from experiments in homing pigeons (*Columba livia*) (Sasaki and Biro, 2017). This study found that chains (“generations”) of pairs in which information was pooled between multiple individuals over five iterations (or “generations”) created routes that were eventually more efficient than the two control chains consisting of solo fliers or pairs that stayed the same. The authors argued from this that collective intelligence in animal groups can initiate CCE (Figure 2). Finally, observational evidence from the cultural evolution of humpback whale song has also been proposed as a contender for non-human CCE (Allen et al., 2018), and we explore this in more detail below.

These examples are however, open to critical scrutiny due to lack of direct evidence of both social learning and innovation (Dean et al., 2014; Whiten, 2018). Arguably, Sasaki and Biro’s (2017) is the most convincing study from this perspective, but the trait it focuses on, a navigational route, could in theory be improved readily by a practicing individual without social input. From a product-focussed perspective on CCE, it is not out of the question in most of non-human examples that an individual could learn to produce the documented trait improvements asocially (Tennie et al., 2020), and from this perspective the migration route example is arguably strongest. Finally, all these cases focus on the refinement of a particular skill rather than an entirely new innovation or recombination (Whiten, 2018).

### Humpback Whale Song Case Study

The roots of our enquiry into the tension between CCE and change in aesthetic cultures was that its resolution has important implications for thinking about whether non-humans have elements of CCE, and in particular, whether humpback whale song should be considered an example, as suggested by Allen et al. (2018).

Some of the strongest evidence for non-human culture is found in the complex songs of humpback whales (*Megaptera novaeangliae)* (Payne and Payne, 1985; Garland et al., 2011). Male humpback whales produce a vocal sexual display called “song” during the breeding season. Song is a long, stereotyped acoustic signal with a hierarchical structure, such that each song is composed of a set of themes, each theme is composed of repeated phrases and each phrase is composed of a stereotyped sequence of units (Payne and McVay, 1971; Suzuki et al., 2006). All male humpback whales of each breeding population sing the same song at any given time. The speed of changes to a song that spread across a population indicates that song sequences are socially learned (Tyack and Sayigh, 1997; Janik and Slater, 1998). Generally, each song changes gradually with all singers of the same population updating their song resulting in the maintenance of similarity across the population (Payne and Guinee, 1983; Payne and Payne, 1985). The transmission of song in the South Pacific Ocean is of particular interest to researchers due to the occurrence of song “revolutions” in which a population discards a current song type in favour of a new, and completely different song type (Noad et al., 2000; Garland et al., 2011). Song types have been found to radiate eastward across the South Pacific Ocean. For example, the song of Eastern Australia was transmitted eastward all the way to French Polynesia in 2 years (Garland et al., 2011).

**FIGURE 3:**
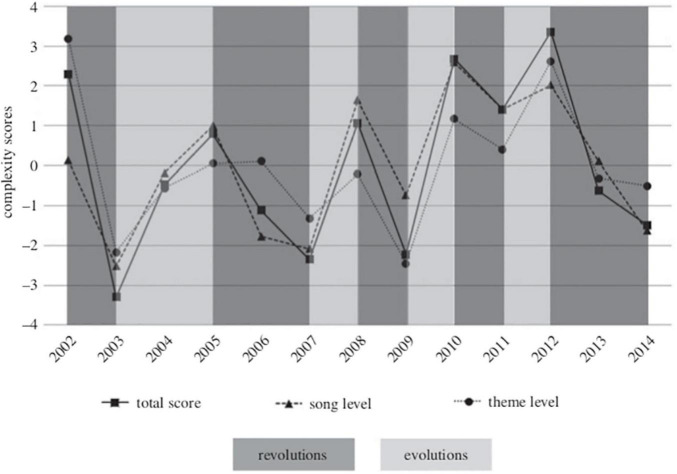
Taken from Allen et al. (2018): “Song complexity scores for each year (2002–2014) representing complexity at the (i) song-level, (ii) theme-level, and (iii) total complexity. Revolution and evolution transitions are demarcated”.

Allen et al. (2018) examined the song structure of humpback whales off the west and east coasts of Australia over thirteen consecutive years. The west coast song regularly spread to the east coast during “revolutions,” but songs underwent more gradual changes in between these events. Allen et al. (2018) found that the complexity of songs, measured as the number of distinct units per phrase and overall song duration, increased as a song evolved between these revolution events (typically over 1–2 years). However, as old songs were replaced with new songs during revolution years complexity was reduced, only to build up again between revolutions (Figure 3). It is thought that an increase in complexity may represent embellishment by males wishing to stand out to females and that reductions in complexity during revolutions may indicate a limit to the social learning capacity of novel material in humpback whales (Allen et al., 2018). Due to the conformity in general song structure at any one time it can be assumed that changes by individual males are incorporated by the population at large and then further built upon to create this incremental increase in complexity over a song’s lifetime.

These cycles of innovation and transmission that produce an increase in complexity mirror the mechanisms described in CCE literature and makes humpback whale song a potential non-human example of CCE. But in what sense has the song “improved”? Is the more complex song “better”? Or is the actual content selectively neutral (borrowing a genetic fitness term for the fitness of cultural traits), only significant within a specific population at a specific time? These questions are also relevant to ongoing debate over the evolution of aesthetics in non-human animals (e.g., see Prum, 2017 and Patricelli et al., 2018). While we do not mean to uncritically compare the experience of humpbacks hearing a song to human aesthetic experiences, it does seem legitimate to ask what basis, if any, do we have to differentiate between “fitness neutral” evolution of song in this example, and change in human aesthetic products?

## Technological and Aesthetic Cultures Evolve in Different Ways

While we do not wish to equate human music with humpback whale song, we do wish to point out that when humpback whale song is examined through the lens of cumulative cultural evolution, the secondary questions posed about improvement in performance are similar to those of aesthetic value (or aesthetic attractiveness) in human aesthetic culture. Humpback whale song in at least some populations increases in complexity through cultural evolution, akin to some changes in musical styles (Percino et al., 2014; Allen et al., 2018). However, this increase in complexity is reduced when song revolutions occur, which would seem contrary to the ratchet effect, or sequential improvement central to the CCE framework. As with the cultural evolution of human art forms, we cannot uncritically equate change (in this case an increase in complexity) with an improvement in performance. There are iterations of embellishment, upon which each individual learns and incorporates changes or additions into their own repertoire, after which the same individual may add further embellishments/changes/additions. In a similar manner, the core processes of learning, innovation, and transmission are present in aesthetic cultural products. Consequently, the key question arises: do we widen our definition of CCE (potentially dropping or weakening one of Mesoudi and Thornton’s core criteria) to encapsulate both aesthetic cultural products in humans and strengthen the case for non-human CCE (e.g., humpback whale song)? Or, if we cannot show that the human aesthetic cultural products improve in any measurable way, do we exclude them both from the CCE framework (for example by rebranding CCE as CTC, as in Osiurak and Reynaud, 2019)?

We have focussed on aesthetic value as the metric by which aesthetic products might or might not be said to improve, but some authors consider advancements in the means of production that create an aesthetic product to qualify as an improvement within the aesthetic domain (Tinits and Sobchuk, 2020). We argue in contrast that while the means of production may be an input of consideration to the overall aesthetic value of an aesthetic product, the means of production cannot solely determine improvement in the aesthetic value or experience of an aesthetic product. Instead, the means of production are cultural traits within the technological domain. The contrasting view from a contemporary study by Tinits and Sobchuk (2020) depends upon the philosophical stance of Becker (2019), a sociologist who argues that art is better understood as a collective activity than as a collection of artworks. According to Tinits and Sobchuk (2020) this means that the mechanisms behind the production of a painting cannot be sharply distinguished from the painting itself (where “painting” could be replaced by a piece of music, a film, a book, etc.). Tinits and Sobchuk (2020) describe cumulative cultural evolution in the aesthetic domain by pointing toward the increase in complexity in the structure of production crews behind films. However, the process by which an aesthetic value arises from the interactions between a product and a specific percipient is complex. Undoubtedly, contextual factors about the process of production (for example knowledge of the circumstances of the production, or the artist’s statements of intent, or a film directors influences) could be important inputs to both the aesthetic experience and the ultimate aesthetic judgement of a given product (Leder et al., 2004). To us, this means that relationship between CCE in production methods and resultant aesthetic values is also likely complex and unlikely to follow simple correlations. Tinits and Sobchuk (2020) present their study as showing CCE in an aesthetic domain, but they are focussed on the means of production (in this case the film industry), which is, at least partially, distinct from the aesthetic value of the final product (the film). In our view, they have shown that the means of production of aesthetic traits can evolve by the process of CCE (traits belonging in the technological domain), rather than showing CCE in the aesthetic traits themselves. Similarly, the nineteenth century expansion of the Western orchestra both in size and variety of instruments would not automatically mean an increase in aesthetic value or attractiveness of the aesthetic products created through such an expansion.

A theory related to Becker’s is held of music by sociologist Christopher Small, who prioritises performance over the musical “work,” and defines “musicking” as the participation in any capacity whatsoever in a musical performance (1998). Musicking is just one aspect of a society’s ritual activities which articulate its (ideal) social relationships, and a participant’s aesthetic pleasure in a performance arises when its musical gestures successfully articulate or affirm these relationships in a way which meshes with the participant’s own view of them. Aesthetic judgements and value, for Small, thus refer implicitly to the society that has given rise to the performance being judged. If the degree of pleasure is related to the degree of fit between performance and participant, it seems highly unlikely that aesthetic value can increase in the open-ended way implied by Tinits and Sobchuk. Our stance is supported somewhat by Yang et al.’s (2019) suggestion that aesthetic experiences do vary across cultures, as aesthetic judgements varied between participants with different cultural backgrounds, with aesthetic judgements more positive when participants viewed visual art from their own culture.

Such detailed analyses of the relationship between the technological and aesthetic domains of cultural evolution can, in our view, help clarify current debates in cultural evolution between advocates of Californian cultural evolution theory and more recent contributions from supporters of the Parisian cultural attraction theory. We have analysed above the ways in which cultural change might differ between technical and aesthetic domains. How might our treatment lead to additional understanding of why Parisian-perspective cultural attraction and Californian-perspective cultural evolution can co-exist? We propose that they are accounts of cultural change that are directed at different types of fitness landscape. In the technological domain, the Californian perspective works well because a problem, once defined, is essentially static – for example: “produce using available materials a human powered craft for navigating sheltered waters in the Arctic” – and solutions can then be objectively compared. In this domain, discussing cumulative cultural evolution in the context of improving performance is relatively unproblematic.

In contrast, within the aesthetic domain, the ‘‘problem’’ -- maximising aesthetic value -- can never be static because the target, the aesthetic value judgements of the percipient, is always moving. Tastes are changing, and sub-groups branch toward radically different and sometimes fundamentally incompatible judgements of value, such that solutions cannot be objectively compared, and can in fact be described as arbitrary with respect to any criterion that does not reside within a human mind. Here, the value of ‘‘solutions,’’ i.e., aesthetic products, is defined as much by the characteristics of the audience as by the nature of the solution, which is why the notion of cultural attractors, features of particular groups of minds at particular times, can be valuable as a tool for explaining cultural change in this domain^10^. We should therefore expect from the arguments we have laid out that fundamentally different principles could govern cultural changes in the two domains, and as a result be mindful of the problems of confusing or conflating the two. Inevitably there are going to be cases where the contrast is not clear cut, but in general we suggest that recognition of this contrast between aesthetic and technical domains provides a conceptual framework in which both Parisian and Californian perspectives on cultural change can and should co-exist.

Our manuscript brings together thought from biology, musicology and philosophy with the aim of disentangling the implications of applying the idea of improvement in performance that is critical to the concept of CCE to the cultural evolution of aesthetic attractiveness or value. Our overall conclusion is that this is clearly not a trivial task and requires more attention than has been previously allocated in the CCE literature, which has been predominantly technological in focus (Vaesen and Houkes, 2021). Depending on the philosophical stance taken, this task may even prove impossible. Through a philosopher’s lens we have examined the nature of aesthetic value and whether it can be measured in any meaningful way, and from the perspective of musicology we have examined a long tradition of thought about whether the aesthetic value of a specific example, music, can progress. Both views find that the answer is not straightforward and importantly that the answer we choose has potentially important repercussions for how we treat an array of cultural phenomena both in humans and other animals. Lastly, we have discussed a non-human animal case study to evaluate the repercussions of our findings on particular cases of non-human animal culture. We hope that our manuscript opens up new avenues of discussion about CCE within the aesthetic domain and that this is just the beginning of a fruitful discussion between disciplines.

The question remains though as to whether cultural change in the aesthetic domain can ever be meaningfully described as cumulative, if there cannot be unambiguous consensus on the nature of what is accumulating? The answer matters. If it is no, which from the perspective of the philosophy of aesthetics is arguably the supported position, and the one we lean toward, cultural change in the arbitrary form of animal signals must be excluded – humpback whale song is not, from this perspective, an example of cumulative cultural evolution. The perhaps uncomfortable extension, however, is that large swathes of human cultural production in the aesthetic domain must also be moved out of the cumulative box, including Mesoudi and Thornton’s (2018) last example of “the aesthetic attractiveness of art”. In contrast, if the answer is yes, then Mesoudi and Thornton’s original examples all stand, but we have no basis for saying that humpback whale song does not also show cumulative cultural evolution, and the philosophical issues raised above become a more serious problem for this account of CCE. We do not pretend to answer this question here definitively, rather our goal has been to articulate it, and the consequences of choosing each answer, and we hope to have clarified how, in our view, accounts of cumulative cultural evolution are currently resting on the horns of a dilemma when it comes to the aesthetic domain.

## Author Contributions

NS and LR conceived the idea. NS contacted discipline experts to begin interdisciplinary dialogue and directed and coordinated the writing. NS, JU, and AS drafted the manuscript. All authors provided comments on multiple manuscript drafts, and read and approved the final manuscript.

## Conflict of Interest

The authors declare that the research was conducted in the absence of any commercial or financial relationships that could be construed as a potential conflict of interest.

## Publisher’s Note

All claims expressed in this article are solely those of the authors and do not necessarily represent those of their affiliated organizations, or those of the publisher, the editors and the reviewers. Any product that may be evaluated in this article, or claim that may be made by its manufacturer, is not guaranteed or endorsed by the publisher.

## Footnotes

1 A common example cited as the pinnacle of human cumulative culture, as beyond the capacities of one individual, is when one person stepped on the moon. This feat was a team enterprise including technologies across a range of disciplines, the research for which was carried out over multiple centuries (Dean et al., 2014).

2 See Sterelny (2017) for a more in-depth analysis of the agreements and disagreements between these two perspectives of thought in cultural evolutionary research.

3 Walton’s (1970) “Categories of Art” contains useful suggestions about which qualities aesthetic experiences of artworks should be directed at.

4 The standard model is not without its detractors, such as Sharpe (2000) and Watkins and Shelley (2012), though neither give a positive account of what might replace it.

5 “Magnitude” is Beardsley’s term for the collective effect which three inter-related factors have on the character of an experience. The factors are: (i) how *unified* – “coherent” and “complete” – the experience is; (ii) how *intense* the experience is; and (iii) how *complex* the experience is (i.e., “the range or diversity of the distinct elements that it brings together into its unity”) (Beardsley, 1958, p. 529) (see Dickie, 1965, 1974; Beardsley, 1969 for discussion of these criteria).

6 Other proponents of the standard model, or a variant of it, include: Ross (1930), Lewis (1946), Dickie (1988), Levinson (1992, 1996), and Goldman (1995, 2006). See Forsey (2017) for a critique of Stecker.

7 “Under some or other of these imperfections, the generality of men labour; and hence a true judge in the finer arts is observed, even during the most polished ages, to be so rare a character: Strong sense, united to delicate sentiment, improved by practice, perfected by comparison, and cleared of all prejudice, can alone entitle critics to this valuable character; and the joint verdict of such, wherever they are to be found, is the true standard of taste and beauty” (Hume, 1757/1995).

8 There is no straightforward or standardly accepted definition of musical complexity, evidenced by the fact that the standard encyclopaedia of music, “Grove Music Online,” contains no entry for the term. However, we note here that “complex,” as antonym of “simple,” is often used informally to describe either an overall impression or various dimensions of music, such as melody, harmony, rhythm, timbre and structure, from at least two points of view: those of the performer and the listener. Naturally, this indexes performance skills and listener familiarity and preferences, and can never be entirely objective. Here Beardsley’s characterisation of the complexity of an aesthetic experience (already cited above) as “the range or diversity of the distinct elements that it brings together into its unity” seems to be a reasonable description of the term’s application to musical works by writers in historical musicology such as Forkel, and for the main part this is how we use it in this section of our manuscript (the exceptions are for the corpus studies cited below by Percino et al. (2014) and Parmer and Ahn (2019), which use information-theoretic measures).

9 “Ce qu’on appelle en général *progrès* n’est que *transformation*”.

10 While Scott-Phillips et al. (2018) discuss “ecological attractors” within the context of cultural attraction, this seems to us a flawed conflation of two fundamentally different domains of cultural change.
