# Supplementary material for: From Beethoven to Beyoncé: Do Changing Aesthetic Cultures Amount to “Cumulative Cultural Evolution?”
Source: Front Psychol. 2022 Feb 9;12:663397. doi: 10.3389/fpsyg.2021.663397 (PMC8864182; doi:10.3389/fpsyg.2021.663397)
Supplement: Supplementary file 1 [file Table_1.DOCX]

Supplementary Material

# Supplementary Figures and Tables

Tables:

Table 1: Examples of 'improvements' in literature as taken from Mesoudi & Thornton's supplementary material A: Human, B: Non-human

A: HUMAN

| **Study / species** | **Improvement** |  |
| --- | --- | --- |
| Caldwell & Millen 2008 | Yes – Towers got higher and planes few farther |  |
| Mesoudi 2008 | Yes – Arrowheads scores increased |  |
| Kirby, Cornish & Smith 2008 | Yes – Increases in learnability of languages |  |
| Beppu & Griffith 2009 | No |  |
| Wisdom & Goldstone 2010 | Yes – Increase in team performance |  |
| Derex, Godelle & Raymond 2013 | Yes – Fishing nets scores increased |  |
|  |  |  |
| Wasielewski 2014 | Yes – Performance of clay and reed devices increased |  |
| Kempe & Mesoudi 2014 | Yes – Number of pieces solved increased |  |
| Muthukrishna et al. 2014 | Image editing task: Yes – Image editing skills increased. Knot tying task: No – Participants could only do worse than the initial demonstrator |  |
| Derex & Boyd 2015 | Yes – New tools and high score totems were produced |  |
| Zwirner & Thornton 2015 | Yes - More efficient baskets were produced |  |
| Derex & Boyd 2016 | Yes – New active ingredients were produced and remedies scores got improved |  |
| McGuigan et al. 2017 | Yes – New and higher rewards were obtained |  |
| Fay et al. 2018 | Yes - Reproduction accuracy increased |  |

B: NON-HUMAN ANMAL

| **Study/ species** | **Improvement** |
| --- | --- |
| Sasaki & Biro 2017 – homing pigeons (Experimental) | Yes |
| Fehér et al. 2009 – zebra fnches (Experimental) | Yes – vocal learning |
| Claidière et al. 2014 – Guinea baboons (Experimental) | Partly – Improvements in individual performance (correctly remembered blocks) across transmission chains, but better performance does not provide any functional benefts to the individual |
| Dean et al. 2012 – chimpanzees and capuchin monkeys, compared to human children (Experimental) | No – Animals typically failed to obtain more desirable rewards (NB there is no evidence that more desirable rewards had greater nutritional value) |
| Marshall- Pescini & Whiten 2008 – chimpanzees (Experimental) | No – Asocial learning of probing by one individual only; no social learning of probing |
| Price et al. 2009 – chimpanzees (Experimental) | Yes – in some conditions, food rewards could only be accessed by putting together components to make a longer tool |
| Yamamoto et al 2013 – chimpanzees (Experimental) | Yes – Some individuals switched to a more effective technique after observing demonstrators (sucking) |
| Vale et al 2017 – chimpanzees (Experimental) | Yes – Learning a more efficient technique following observation of a trained demonstrator (though some individuals also learned to modify tools in the absence of a demonstrator) |
| Davis et al 2016 – chimpanzees (Experimental) | Yes – Some individuals, forced by experimental design to use a highly inefficient method switched to a more efficient (faster) method after observing demonstrators |
| Kendal et al 2009 – nine- spined sticklebacks (experimental) | Yes – switching to the rich patch provides foraging returns |
| Schofeld et al 2017 – Japanese macaques (Observational) | No – But novel behaviours (e.g. digging pools to wash food) argued to build on previous socially learned behaviours |
| Hunt & Gray 2003 New Caledonian crows (Observational) | No – No evidence that different tools differ in their efficacy |
| Filatova et al. 2013 – Killer whales (Observational) | No – Vocalisations do not become “better” in any functional sense. There may be cultural change (e,g, through processes analogous to drift), but not CCE according to our defnition. |
| Boesch 2003; Sanz et al. 2010 and others – wild chimpanzees (Observational) | No – No direct evidence that supposedly complex tools are more effective than simpler tools |
| Perry et al. 2011 – wild capuchin monkeys (Observational) | No – no evidence that new behaviours provide fitness benefts |

Published by the Royal Society under the terms of the Creative Commons Attribution License <http://creativecommons.org/licenses/by/4.0/>, which permits unrestricted use, provided the original author and source are credited.
